# Recommendations for designing conversational companion robots with older adults through foundation models

**DOI:** 10.3389/frobt.2024.1363713

**Published:** 2024-05-27

**Authors:** Bahar Irfan, Sanna Kuoppamäki, Gabriel Skantze

**Affiliations:** ^1^ Division of Speech, Music and Hearing, KTH Royal Institute of Technology, Stockholm, Sweden; ^2^ Division of Health Informatics and Logistics, KTH Royal Institute of Technology, Stockholm, Sweden

**Keywords:** participatory design, co-design, human-robot interaction, companion robot, open-domain dialogue, foundation models, large language models, elderly care

## Abstract

Companion robots are aimed to mitigate loneliness and social isolation among older adults by providing social and emotional support in their everyday lives. However, older adults’ expectations of conversational companionship might substantially differ from what current technologies can achieve, as well as from other age groups like young adults. Thus, it is crucial to involve older adults in the development of conversational companion robots to ensure that these devices align with their unique expectations and experiences. The recent advancement in foundation models, such as large language models, has taken a significant stride toward fulfilling those expectations, in contrast to the prior literature that relied on humans controlling robots (i.e., Wizard of Oz) or limited rule-based architectures that are not feasible to apply in the daily lives of older adults. Consequently, we conducted a participatory design (co-design) study with 28 older adults, demonstrating a companion robot using a large language model (LLM), and design scenarios that represent situations from everyday life. The thematic analysis of the discussions around these scenarios shows that older adults expect a conversational companion robot to engage in conversation actively in isolation and passively in social settings, remember previous conversations and personalize, protect privacy and provide control over learned data, give information and daily reminders, foster social skills and connections, and express empathy and emotions. Based on these findings, this article provides actionable recommendations for designing conversational companion robots for older adults with foundation models, such as LLMs and vision-language models, which can also be applied to conversational robots in other domains.

## 1 Introduction

Robots in elderly care are increasingly targeted towards not only fulfilling practical needs, such as medication reminders or physical assistance, but also as companions to prevent and mediate loneliness through offering social and emotional support in their everyday lives, thus enhancing the psychological wellbeing of users (Banks et al., 2008; Robinson et al., 2013; Pu et al., 2018). Research in companion robots for older adults focused primarily on pet robots, such as PARO (a seal-shaped robot), that do not have natural language processing (NLP) or generation capabilities (Pu et al., 2018). One of the underlying reasons is the limitations in NLP technology, leading to heavy reliance on humans to control robots, either through telepresence or the Wizard of Oz technique (Kelley, 1984) to give the illusion that the robot is autonomous, or by rule-based architectures that allow one-way transactional (e.g., providing medication reminders) interactions or small talk that are not suitable for daily dialogues with older adults.

The recent introduction of “foundation models”, i.e., deep learning models, such as BERT (Devlin et al., 2019), GPT-3 (Brown et al., 2020), DALL-E (Ramesh et al., 2021), and CLIP (Radford et al., 2021), that are trained on broad data (often through self-supervision) that can be applied in or adapted to a wide range of downstream tasks, transformed the scope of what is achievable in many robotics applications (Bommasani et al., 2022). Most prominently, large language models (LLMs) enabled the development of companion robots with social skills due to their ability to process and produce language in an open-domain manner, without restriction on topics or concepts. Recent work incorporated LLMs for open-domain dialogue with robots in therapy (Lee et al., 2023), service (Cherakara et al., 2023), and elderly care (Irfan et al., 2023) domains, revealing their strengths and weaknesses in multi-modal contexts across diverse application areas. These studies underscore the versatility of LLMs in facilitating human-robot interaction (HRI).

Integrating LLMs into human-robot interaction requires awareness of the user’s perceptions, needs, and preferences to ensure that these robots are aligned with human values and can successfully be employed in real-life contexts. Alignment techniques like reinforcement learning with human feedback can improve some model capabilities, but it is unlikely that an aggregate fine-tuning process can adequately represent the full range of users’ preferences and values (Kirk et al., 2023). Participatory design (co-design) approaches enable incorporating these aspects into the design process of robots, through focus groups, interviews, concept generation and design activities, prototyping, and interactions with designed robots (Šabanović, 2010; Frennert and Östlund, 2014). These studies investigate HRI as a relational and social phenomenon, where contextual factors and longitudinal effects alter the interactions with the robot (Bradwell et al., 2020; Søraa et al., 2023). An emphasis is based on how robots are shaped in interaction with the wider socio-cultural and physical environment into which robots are introduced. Against this background, it becomes important to explore the shared understanding of companion robots for open-domain dialogue, which influences the expectations, values, norms, and possible contradictions that older adults have towards companion robots.

**FIGURE 1:**
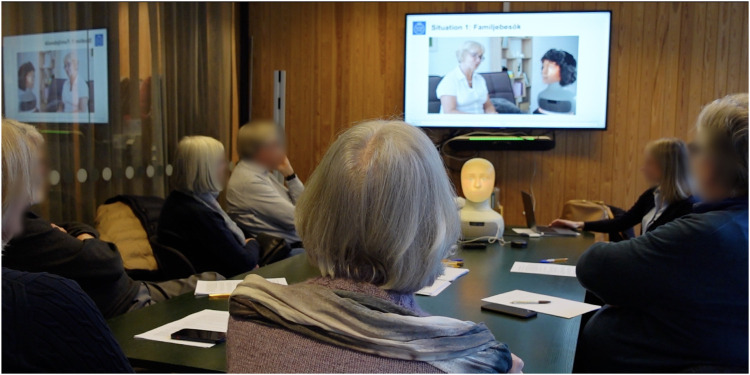
Participatory design workshop with older adults.

This study investigates older adults’ expectations towards conversational companion robots to provide social and emotional support in their daily lives, and provides design recommendations on how to achieve these expectations through foundation models. Participatory design workshops were conducted with 28 Swedish-speaking older adults, aged 65 and over (Figure 1). The workshops involved a demonstration of open-domain dialogue with an autonomous Furhat robot employing an LLM (GPT-3.5 text-davinci-003), and (6-8 participant) focus group discussions deriving from conversational design scenarios that can occur in their everyday lives. The contributions of this article encompass two key aspects:

1. Through a qualitative approach, identifying socially shared expectations of older adults regarding conversational companion robots for everyday life (Section 4, summarized in Table 1),
2. Formulate actionable design recommendations for integrating foundation models into these robots to meet these expectations, focusing on LLMs for their advanced linguistic capabilities, combined with vision-language models and other state-of-the-art technology for multi-modal aspects (Section 5).

**TABLE 1:** Older adults’ expectations towards companion robots for open-domain dialogue.

Main category	Subcategory	Contextual example
Active listening	Eliciting information from the user through follow-up questions	Urging the user to tell their concerns in loneliness
	Inspiring the user to think positively	Encouraging the user to think of something funny for overcoming boredom
	Facilitating self-reflection	Asking for explanations on the user’s (negative) perceptions and thoughts
Passive listening	Registering information from social events	Listening quietly to group conversations during a game with friends, and talking to the user about it when alone
	Debriefing and reminiscence	Providing awareness for the user (e.g., on their relationship with others) based on their conversations with friends
Personalization	Learning and referring to details in previous conversations	Remembering names and ages of family members, and shared history
	Providing advice based on situational context	Giving suggestions about grandchildren
	Providing recommendations based on user preferences	Suggesting a movie
	Forming a relationship	Having a personality and stating own opinions based on shared history
Privacy protection and data control	Preventing others from accessing user data	Identifying the user prior to conversations
	Ensuring confidentiality of personal data	Using embedded systems or privacy-preserving frameworks for cloud-based services
	Allowing users to delete learned information	Forgetting difficult situations with family members, or deleting incorrectly provided information
Information retrieval	Reminding daily agenda	Mentioning the doctor’s appointment on the day
	Taking initiative in providing daily information	Reporting weather and news in the morning
	Explaining contextual information for social and emotional support	Interpreting a doctor’s diagnosis and recommending ways to help
	Enabling fact-checking through natural language communication	Asking for facts based on a disagreement during a game with friends
Social connectedness	Connecting with other people to counter loneliness	Offering to call family members or friends, or finding new connections online
	Strengthening communication skills of older adults over 90	Encouraging conversation to retain vocal articulation when living alone
	Supporting maintenance of cognitive skills for adults with dementia	Singing with the user, or encouraging them to sing
	Engaging in leisure activities together	Watching television together and discussing content
Emotional expressiveness	Responding empathetically	Expressing joy (e.g., celebrating becoming a grandparent) or sorrow (e.g., mourning a loss) verbally
	Changing voice intonation based on the context of the conversation	Sounding happy when congratulating the user for being a grandparent
	Having contextual facial expressions	Laughing and smiling when appropriate

## 2 Background

### 2.1 Companion robots for older adults

Companion robots are socially assistive robots that are designed to respond to the social, emotional, and cognitive needs of older adults and enhance their quality of life, activity, and participation. Studies involving companion robots are focused on the acceptance and use among older adults and caregivers in organizational contexts (e.g., Heerink et al., 2008; Pino et al., 2015), therapeutic effectiveness of companion robots to agitation and anxiety (e.g., Bradwell et al., 2020), and design features of companion robots to promote dignity and autonomy (e.g., Coghlan et al., 2021). A high level of individual differences in willingness to interact and establish a relationship with the companion robot has been observed in older adults (Thunberg et al., 2021). Their acceptance is influenced by functional variables related to social interaction (Heerink et al., 2010), as well as age-related perceptions of their self-image and user-image (Dudek et al., 2021), and individual values and aspirations (Coghlan et al., 2021). Robinson and Nejat (2022) provide a recent overview of the robot types and features used in socially assistive robots for senior care.

Design features of companion robots should reinforce older adults’ autonomy, dignity, and skill level, which often remains a challenge in robot design (Kuoppamäki et al., 2021). Participatory design (co-design) has been proposed as a solution to design more inclusive and suitable companion robots for older adults, and to promote mutual learning between participants and researchers (Lee et al., 2017; Kuoppamäki et al., 2023). This approach takes participants’ self-perceived thoughts and opinions into consideration and highlights factors that influence their attitudes towards robots in developing robot concepts, applications, and interaction modalities. These studies make use of interviews and focus group discussions after having been shown pictures or videos of companion robots (e.g., Søraa et al., 2023), and empirical material collected in real-world settings where older adults get to engage with companion robots for short or longer period of time (e.g., Chang and Šabanović, 2015; Ostrowski et al., 2021).

Due to the lack of a robust solution for open-domain conversation (i.e., conversations that are not limited to any topics) that can arise in the daily lives of older adults, most prior studies that provided recommendations to design companion robots for older adults focused on non-conversational aspects based on robot pets (e.g., Lazar et al., 2016; Bradwell et al., 2019). Only a few studies touched upon the potential for conversational aspects, however, these recommendations did not explore beyond one-way transactional interactions, such as providing medication reminders, exercise, entertainment, and motivation, rather than mutual everyday conversations (e.g., Lee et al., 2017; Thunberg and Ziemke, 2021; Søraa et al., 2023). Other work solely outlined desired high-level functionality rather than providing actionable solutions (e.g., algorithms) on how to achieve the expectations of older adults for companion robots (e.g., Pino et al., 2015; Deutsch et al., 2019; Lin et al., 2020; Bradwell et al., 2021; Gasteiger et al., 2021). In this work, we gather expectations of older adults towards conversational companion robots based on focus groups deriving from everyday situations, in addition to providing concrete suggestions on achieving the desired functionality based on foundation models, such as LLMs and other state-of-the-art architectures, which does not exist in prior work.

Similarly, despite the numerous studies investigating the use of conversational companion robots with older adults, only a few studies have employed autonomous conversational robots for open-domain dialogue (e.g., Sorbello et al., 2016; Kuo et al., 2021; Ostrowski et al., 2022; Khoo et al., 2023). Other studies focused on task-oriented dialogue that gives reminders, answers questions, provides weather reports, and plays games with this age group (e.g., Khosla and Chu (2013); de Graaf et al. (2015); Carros et al. (2020). The earliest study that involved an autonomous conversational robot for older adults was that of Yamamoto et al. (2002). The robot was able to recognize 300 Japanese words for daily greetings and functional commands with 47% accuracy, and respond accordingly. It was evaluated with 7 older adults on an average of 62 days. In contrast, current speech recognition systems can mostly accurately recognize more than 100 languages, with 70%–85%^1^ accuracy in adult speech (Irfan et al., 2021a) and 60%–80% in children’s speech (Kennedy et al., 2017). All task-oriented dialogue studies used rule-based architectures (i.e., pre-written templates for input and output responses), and only one of the open-domain dialogue studies integrated foundation models (LLMs) into a companion robot (Khoo et al., 2023). Only one study applied co-design in the development of autonomous conversational robots with older adults (Ostrowski et al., 2021). In contrast, our study integrates a foundation model (LLM) into the robot to guide participatory design with older adults and offers corresponding design recommendations to meet those expectations in conversational companion robots.

In real-world applications of companion robots for older adults, there are only a few that are available for purchase, such as non-conversational robot pets (PARO robot seal^2^ and Joy for All cat and dog robot toys^3^) and ElliQ conversational desktop robot with a screen (Intuition Robotics^4^, only available for US customers). To alleviate loneliness, ElliQ proactively provides daily reminders and check-ins for health measures, gives news, weather and sports updates, makes small talk, encourages connection with family and friends, plays music, and offers games and trivia for older adults. It learns from user interactions to personalize its suggestions. However, it is unclear how this learning occurs due to proprietary software, which is updated every 3–4 weeks (Broadbent et al., 2024). The robot was deployed to older adults across 15 programs from various healthcare organizations in the US and Canada since its release in 2022. A study with 173 users who used the robot over 30 days showed that 80% agreed to feel less lonely with the robot. However, despite the effectiveness of proactivity in addressing loneliness (Ring et al., 2013), some users were surprised or annoyed by the proactive features (Broadbent et al., 2024). Other studies supported the negative perceptions of proactive features of the robot, such as being perceived to be talking a lot, threatening their independence, lacking compassion, and being rude, invasive, intrusive, or patronizing (Deutsch et al., 2019; Coghlan et al., 2021).

Previous studies have shown that robots can help combat loneliness in older adults as companions or catalysts for social interactions (Gasteiger et al., 2021). User’s self-perceived loneliness (defined as a subjective experience of lack of social connectedness with other people (Newall and Menec, 2019)) is also positively associated with willingness to buy a robot companion (Ghafurian et al., 2021; Berridge et al., 2023). Nonetheless, older adults tend to think that a companion robot cannot make them feel less lonely (Berridge et al., 2023). These studies, however, have been limited to companion robots with limited or lack of capabilities for having a (open-domain) dialogue with a human. In this study, we analyze older adults’ reflections on conversational companion robots’ roles in their daily lives to provide social and emotional support and alleviate loneliness.

In addition to robots, spoken dialogue agents, such as Amazon Echo, and embodied conversational agents (i.e., virtual agents) that provide task-oriented interactions and small talk were shown to address loneliness in older adults (Loveys et al., 2020; Gasteiger et al., 2021; Jones et al., 2021). However, speech recognition errors and unfamiliarity with spoken dialogue systems (e.g., using a wake word and transactional commands) created adverse user reactions. Older adults did not find valuable use cases for these systems, and considered them as toys, with limited conversational capabilities being the most critical challenge in these systems (Trajkova and Martin-Hammond, 2020; Kim and Choudhury, 2021). In addition, there is extensive literature that shows the benefits of robotic embodiment in improving user perceptions of the agent (Deng et al., 2019). Thus, this study focuses on a companion robot with open-domain dialogue capabilities.

### 2.2 Foundation models in conversational agents

Prior research initially focused on BERT (Devlin et al., 2019) for dialogue state tracking, intent classification, and response generation (e.g., Dong et al., 2019; Tiwari et al., 2021) primarily in task-oriented dialogue, which is designed for a specific goal, such as restaurant booking. Recently, LLMs (e.g., GPT-3 (Brown et al., 2020), LLaMA (Touvron et al., 2023), Falcon (Penedo et al., 2023), Pythia (Biderman et al., 2023), Mistral (Jiang et al., 2023)) that are trained on vast amounts of textual data, showed promise for generating coherent text and speech by using prompts for inferring the context, thereby, enabling open-domain dialogue with unrestricted topics (Huang et al., 2020). Traditionally, LLMs have been employed within text-based chatbot systems, article generation, code generation, and copywriting (Zhao W. X. et al. (2023) provide an extensive survey of LLMs). On the other hand, multi-modal LLMs (e.g., GPT-4 (OpenAI et al., 2023), Gemini (Reid et al., 2024), see (Li C. et al., 2023) for a review) combine text with audiovisual features to provide end-to-end solutions for dialogue generation in agents.

To date, very few studies have empirically investigated users’ experiences of interacting with LLMs in a companion function for social and emotional support. Ma et al. (2023) explored the benefits and challenges of using LLMs (ChatGPT) for mental wellbeing with a conversational agent to help decrease loneliness by generating friendly or empathetic responses that simulate a conversation with a human therapist. Perceived benefits were increasing accessibility to therapists and the opportunity to receive non-judgmental support in therapy, in addition to improving self-confidence and promoting self-reflection and self-discovery. The main perceived challenges included harmful content, limited dialogue memory capacity, inconsistency in communication style, concerns about dependency on LLMs for mental wellbeing support, and the associated stigma of seeking such support from a virtual agent. For enhancing a user’s wellbeing, a key aspect of the companionship of an agent is to foster closeness, such as trust, warmth, and understanding. This involves sharing personal information, providing support, and engaging in joint activities, all facilitated by verbal and non-verbal cues, like empathy, humor, encouragement, and politeness (Loveys et al., 2022). Jo et al. (2023) leveraged LLMs for a public health intervention in an open-domain chatbot to support socially isolated individuals (middle-aged adults) through check-up phone calls. Users perceived that the system helped mitigate loneliness and provided emotional support through empathetic questions about their health, hobbies, and interests. However, it was perceived as impersonal due to the lack of follow-up questions on past conversations.

While various foundation models are used in robotics for manipulation, navigation, planning, and reasoning (Xiao et al., 2023), only LLMs are used in the context of conversational robots. For instance, LLMs have been used for developing conversational robots with empathetic non-verbal cues (Lee et al., 2023), giving adaptive presentations (Axelsson and Skantze, 2023), functioning as a receptionist (Cherakara et al., 2023; Yamazaki et al., 2023), and supporting wellbeing of older adults (Khoo et al., 2023). Khoo et al. (2023) is the only study that integrated an LLM (fine-tuned GPT-3) into a companion robot for open-domain dialogue with (7) older adults, in addition to our prior work (Irfan et al., 2023). Most participants in that study found the interaction with the robot enjoyable, felt comfortable with it, and perceived it as friendly. However, the individual willingness to use the robot varied among participants, with some suggesting that it might be more suitable for older adults with dementia. However, the study did not incorporate older adults’ perspectives on applying LLMs to companion robots through a co-design approach. In our prior study (Irfan et al., 2023), we investigated the challenges of applying LLMs to conversational robots, deriving from the one-on-one interactions of a robot with LLM with older adults, that were conducted after the discussions in the design scenarios. The challenges were found to be affected by the multi-modal context of conversations with robots that go beyond the textual linguistic capabilities of LLMs, leading to frequent interruptions, repetitive and superficial conversations, language barriers, and confusion due to outdated and incorrect information. In contrast, in this work, we investigate the expectations of older adults using thematic analysis of the focus groups, followed by design recommendations to apply these expectations to conversational companion robots with foundation models.

## 3 Data and methods

We conducted four participatory design workshops with 28 older adults, aged 65 and over, at the university premises. Each workshop involved 6-8 older adults and lasted 2 h. A Furhat (Al Moubayed et al., 2012) robot employing an LLM (GPT-3.5 text-davinci-003) in a zero-shot fashion, as described in our prior work (Irfan et al., 2023), was used to foster focus group discussions centered around design scenarios that represent situations from older adults’ daily lives. Acapela^5^ text-to-speech engine in Swedish (Emil22k_HQ) was used for the robot’s voice, and the speech rate was decreased to 80% to facilitate understanding among older adults. In order to understand older adults’ expectations and preferences for companion robots such that we can design robots that align with them, rather than them aligning with the technological limitations of current systems, the robot was only demonstrated autonomously for a brief period (2 min) by a researcher. In other words, the participants did not interact with the robot directly prior to or during the focus group discussions to prevent any biases due to technological limitations.

The study protocol consisted of introducing the study, demonstrating the robot’s capabilities for open-domain dialogue, presenting conversational design scenarios with older adults through videos, and facilitating participants’ expectations, needs, feedback, and shared understandings through focus group discussions to outline design recommendations for companion robots. All workshops were documented with video and audio recordings, and focus group discussions with participants were transcribed to text.

The participants were informed that the study aimed to acquire their feedback, insights, and opinions for developing a companion robot, and were encouraged to share both positive and negative perceptions of the robot for social and emotional support. The robot’s capabilities were demonstrated by a researcher talking to the autonomous robot in an open-domain fashion for 2 min. While the robot’s responses changed slightly due to the LLM (which can generate different responses at run time) in each workshop, the researcher led the conversation to the same topics: participatory design workshop with older adults for designing a companion robot, the robot’s thoughts on robots, and their use in elderly care.

### 3.1 Design scenarios

**FIGURE 2:**
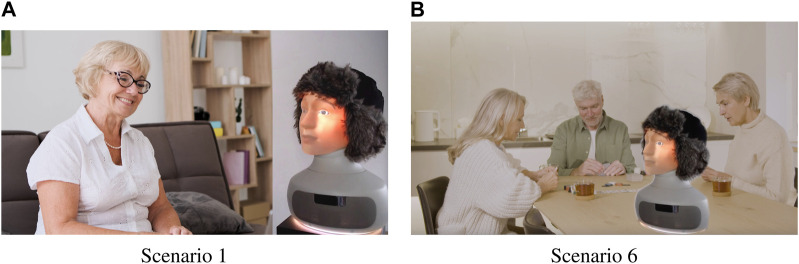
Examples of the design scenarios: **(A)** Coming home after a family visit, and **(B)** friends visiting.

We prepared six design scenarios that were demonstrated to participants with images and videos during the workshop^6^. Videos were adapted based on royalty-free stock video footage^7^ that do not contain audio or text. In two of the scenarios (S1 and S6), a Furhat robot was integrated into the video^8^, as shown in Figure 2, to provide better situational context on the robot interaction, whereas for the rest of the scenarios, the participants were asked to imagine talking to the robot after about the described event. Scenarios represented common situations that older adults could face in their everyday lives:

S1 An older woman returning home after family (children and grandchildren) visit and talking to the robot about it (Figure 2A).

S2 An older woman sitting in a kitchen alone and looking sad.

S3 An older woman talking on a phone and hearing bad news.

S4 An older woman finds out that her daughter is pregnant.

S5 An older man waking up from bed.

S6 Older adults (two women and a man) playing a card game around the table, where the robot was placed (Figure 2B).

After each scenario, participants were asked questions that were adapted from the Likert scale questions in the Almere model (Heerink et al., 2010) and privacy scale (Malhotra et al., 2004; de Graaf et al., 2019):

S1 Would your family members find the robot fascinating or boring? (*Social influence*).

S1 Would you like the robot to remember your conversation? (*Privacy concern*).

S2 Do you think the robot could help you reduce or strengthen the experience of loneliness? (*Usefulness*).

S3 Do you think the robot could personalize social conversation? (*Adaptiveness*).

S4 Do you think the robot could robot give you empathetic support? (*Usefulness*).

S5 Do you think the robot could be a nice conversation partner? (*Sociability*).

S6 Are you worried about your privacy with the robot? (*Privacy concern*).

In addition, the participants were asked, “What kind of conversation(s) would you like to have with the robot in this situation?” and “What would you like the robot to say/talk about?” for each scenario except for the final scenario involving interaction with friends, for which they were asked, “How would you like the robot to interact with you and your friends?”. All questions were followed by “why/how/what” based on the participants’ responses, aimed to initiate the discussions in a semi-structured format, leading to open-ended discussions.

Questions asked in the focus group discussions were centered on participants’ perceptions and expectations of using the robot in envisioned social situations and for the provision of social and emotional support. There were no questions regarding whether or not the participants had experienced loneliness themselves. Therefore, the corresponding discussions represent participants’ reflections about using the robot for loneliness prevention among healthy older adults, rather than investigating the effects of using the robot to reduce loneliness.

### 3.2 Focus group discussions

The presentation of each scenario resulted in vivid discussions in the group, where participants contemplated possible conversational scripts with the robot and shared their first impressions about the companionship function of the robot. Design scenarios were used as an elicitation tool for acquiring “tacit knowledge’” that often may remain hidden and unspoken in social situations (Van Braak et al., 2018). The researchers presented questions to understand the participants’ preferences or self-identified needs and first impressions about the robot for providing social and emotional support in a particular social environment and context. The researchers only contributed to the group discussions when the participants asked them about the capabilities of the robot regarding their suggestions, in which case they responded affirmatively to avoid biasing them with the limitations of the current technology. Focus group discussions lasted approximately 60 min.

### 3.3 Participants

The participants were recruited through an invitation that described the goal of the project and the activities that will be part of the design workshops. Adults aged 65 or older were invited to participate in the invitation, without requiring prior knowledge of robots. The invitation was tailored towards recruiting participants interested in contributing to the development of a companion robot: “Your participation contributes to knowledge about the benefits of this technology for older adults, and you have the opportunity to influence future solutions.” The invitation was distributed via our university’s communication channels, social media, and platforms for gathering senior citizens.

Based on the recommended number (3-4 groups) and size (6-8 participants per group) of focus groups in the literature (Krueger and Casey, 2014), we recruited 28 participants (15 females, 13 males) from the age group 65 and over. All participants were Swedish speakers aged 66 to 86, with an average age of 74.5 (*SD* = 5.6). The majority (22) of the participants were living with a partner, and most (23) did not have prior experience with robots. Participants were offered a small compensation (100 SEK gift card) at the end of the study. The distributed invitation for the study mentioned that a gift card would be given as compensation, but did not specify the amount. The participants were divided into 4 groups, each involving 6-8 people, with an equal number of male and female participants to facilitate an equal presence of opinions from both genders. Since the participants’ opinions or suggestions may influence the group’s opinion as a whole in the focus groups, it is not possible to analyze gender effects. Before starting the study, all participants were given research subject information guided by the Ethical Review Authority, and they gave informed consent for participation in the study and publishing anonymized extractions from the data.

### 3.4 Analytical approach

All focus group discussions were transcribed to text and analyzed using a qualitative thematic analysis method (Hsieh and Shannon, 2005). In the first stage of the analysis, all transcriptions were read through by two researchers in order to form a holistic understanding of the data. In the second stage, one of the researchers used an inductive approach to form themes, iterating over the transcripts multiple times, allowing participants’ self-identified statements to be the basis of the thematic categorization. In the final stage, all statements responding to the main themes were classified thematically, allowing both researchers to collaboratively validate the thematic categories developed in the second stage. The analysis focused on exploring the variance and richness of participants’ insights and opinions.

## 4 Findings

Deriving from the thematic analysis of the focus group discussions, we investigate older adults’ socially shared expectations regarding conversational companion robots, as summarized in Table 1.

### 4.1 Active listening

Discussions around all scenarios were centered around the robot’s ability for ‘active listening’ when the user is alone. That is, the robot should be engaged in the conversation, understand its context, and ask follow-up questions to the user. Robots should be able to “elicit” interaction with users in a way that users feel comfortable with sharing possible concerns beyond just superficial small talk. This could occur in the form of follow-up questions:

But it has to ask a lot of questions so that this lady in this case (referring to the loneliness scenario) gets to talk out her concerns and put everything into words and such. So that the robot becomes good at eliciting that story. *(G1, P2, male)*

Follow-up questions from the robot could inspire the user to think differently about their personal situation, and provide encouragement, by adding “something new, that I have not thought of to make it interesting” (*G3, P1, male*). The robot can also foster a fresh perspective and encourage users to self-reflect:

If you say “I’m bored today”, and then the robot says like this, “Yes, I understand, but yes, is there something funny you can think of?”, or something like that. I do not know, but there will be a lot of follow-up questions and I do not think the robot itself can come up with that much input, but can ask more like this, “What do you mean then? Explain further.”, something like that. Therapy call. *(G1, P2, male)*

### 4.2 Passive listening

On the other hand, when the robot is placed in a social environment, such as a part of a group discussion where people play cards together (scenario 6), the robot should listen “passively”, that is, without reacting to the conversation. However, the robot should still comprehend the information in the conversations, and refer to shared history when the user is alone:

Robot must be able to register, in order to then be able to talk about what he has experienced (−) But not that he should be part of the talks. *(G1, P1, male)*

Then you can talk to (the robot) afterwards, “Was it fun yesterday?”. *(G1, P1, male)*

The participants associated the robot to be a companion in one-on-one interactions, rather than in group interactions, because “When I have my friends at home, I think it should be quiet” (*G1, P5, male*). However, the robot’s ability to provide information from these group settings was considered as appealing and a possibility for debriefing and reminiscence:

You kind of want to talk a little about the evening. What was said, how did you react to it, and sort of reflect on what someone has told you and perhaps vent about jealousy or other things that may arise. So the point is also to be able to debrief in some way, I think. *(G1, P5, male)*

### 4.3 Personalization

Personalization was a recurrent theme in the discussions around all scenarios, brought about by the demonstration of the robot during the design workshop, which indicated that the robot could learn from previous interactions with the user and refer to them in conversation (Irfan et al., 2023). Participants associated this ‘learning’ with understanding their preferences and relationships, such as learning their needs and hobbies, remembering the names and ages of their family members, their residences, and occupations. Initially, the robot needs to learn these details actively through questions, and then refer to them over time:

Either the robot knows a lot about your family or not, so what do you want him to say, if we are completely unknown to each other, do you want him to ask about the family, how it was, and how you feel, but another situation is if he knows all this. *(G3, P2, female)*

If it is a family visit, then you probably want to be asked if there are grandchildren there, for example, how they are doing. Has anything funny happened to them in the forecourt? So that you kind of get a little curious about everyday life. *(G1, P5, male)*

You can tell the (robot) I’m going to see my brother when he’s in the hospital or something like that, and maybe he’ll want to ask how is he? How was it there, how is he doing or something. If you are now going to teach it. *(G1, P5, male)*

Based on the learned information, the robot was expected to provide advice given the situational context:

If the robot has learned about one’s family, it can talk about how to deal with the grandchildren and little things like that. But that presupposes that one, it gets taught quite a lot about a specific situation. *(G1, P2, male)*

Because the robot is framed as a social companion, the participants expressed high expectations towards the robot, including the robot’s ability to do user modeling to understand their personal taste and make recommendations (e.g., on movies), as well as being up-to-date on weather and political events:

If it knows my taste and such, I would say “Can you suggest a good film” for example, it could suggest a good film, which one could see. *(G3, P4, male)*

I have thought a lot about the fact that it says personal robot, do you mean that it should be someone who knows what I think and so on and should also be able to answer what the weather is like ( .. ). It should be sort of up-to-date and it should still know perhaps what I have and think, and then it is personal to me. *(G3, P6, female)*

Participants described the process of ‘personalization’ of the robot in relation to human-human relationships that evolve over time through the mutual sharing of personal experiences and information with each other. If the robot is given a companionship role, it should be perceived as being “as good as any other human” (*G3, P5, male*), with a personality and own opinions, and should be “trained to catch important things” (*G4, P2, male*):

But when you meet other people, it’s not just a matter of talking about your interests and yourself, the interesting thing is just “Yes, but what do you say, do you think so?”. That is to say, we will make very high demands on the robot, I think because, if we can choose to be able to replace it, it will be as good as another human. *(G3, P5, male)*

### 4.4 Privacy protection and data control

Security features, such as user identification, that prevent the robot from sharing the data with others might encourage users to feel more relaxed in the presence of the robot. Otherwise, the participants might feel the need to “censor yourself all the time” (*G3, P2, female*).

If it is stolen, it must have a password or something like that. *(G1, P4, male)*

(There needs to be) some certainty is that it senses who it is talking to. *(G1, P2, male)*

So that it can somehow identify. (*G1, P6, female*)

You are in a retirement home there, and you say “You were with Karin and spoke to her just now, what did she say then?”. *(G3, P4, male)*

It will be very strange if you tell about your whole life and then someone else can access it. *(G3, P3, female)*

In addition, the confidentiality of personal data when cloud-based services are used is a valid concern among older adults, since their data can be used beyond their consent, in addition to being accessed by governmental entities for surveillance, or being open to hacker attacks. Thus, privacy-preserving frameworks should be used when using cloud-based systems. Otherwise, data storage, extraction, and dialogue generation systems should be embedded on the robot.

But how is the robot connected externally, is there … does it have an external connection and different information channels and so on to be able to give feedback to when we talk to it? And then it can go the other way. *(G1, P1, male)*

It can eventually lead to what? A closed society, surveillance, nobody says anything. *(G3, P5, male)*

No, but it is connected. If it is connected to the internet, it can actually be hacked as well. *(G1, P4, male)*

Users should have the possibility to easily delete previous conversations with the robot to facilitate a sense of privacy, especially when there are “difficulties with family members, you may not have a good relationship at all” (*G2, P3, female*), in addition to self-provided (embarrassing or incorrect) information:

If you say something stupid that you regret having said, you can say “Forget it, delete it”. Yes, it is important. *(G2, P4, male)*

### 4.5 Information retrieval

Participants associated the companion robot with many practical informational needs, such as a reminder of the doctor’s visit and daily agenda (for the fifth scenario): “I can tell him my agenda for all the next few weeks, and then he remembers, and then each morning: ‘You have to do this and that today.’” (*G4, P2, male*). These kinds of conversations with the robot would fulfill both practical and social functions, because the users would receive personalized recommendations and reminders along with their daily schedules: “Don’t forget you have to see the doctor at one o’clock” (*G1, P1, male*). Participants also expected the robot to take the initiative to provide information:

“Good morning, today the weather is like this” and “Do you want to hear the latest news”, and things like that. *(G3, P6, female)*

Companion robots can also provide information and explanations on situated contexts, rather than relying on search engines:

If I got bad news (referring to the third scenario), then I would like the robot to tell me what it means, this bad news, it means that this person is going to die within a week, six months, a year. What can I do to help this person? *(G1, P3, female)*

I think of my mother then, who is ninety-four, she has a lot of company from television (−). If (the robot) can answer questions, give factual answers, sort of like you do not have to go to Google but you get those answers from the robot. Then I think it would fulfill a function, like just because you talk about it snowing outside. *(G3, P1, male)*

Users already have many technologies available for information seeking (e.g., phones, computers, spoken dialogue systems), and a companion robot could complement traditional information seeking by providing personalized statements or opinions on the facts through natural language communication. Participants imagined ‘double-checking facts’ with the robot and acquiring its opinions: “You can use it if you disagree on some factual issue (referring to the sixth scenario for playing a game with friends) (−) ‘Leo (robot), what do you say, what do you think?’” (*G3, P3, female*). This way, the robot could become handy because “you do not have to pick up your mobile phone” (*G3, P3, female*).

### 4.6 Social connectedness

During the discussions of scenarios 2 to 4 (loneliness, hearing bad and good news), participants appeared comfortable talking about their emotions with the robot. Expressing or sharing emotions with the robot was primarily associated with experiences of loneliness. Participants considered it beneficial to disclose their experiences of loneliness with the robot, for which the robot could provide advice on how to get in touch with other people. In these situations, sharing emotions was perceived as a necessity for receiving emotional support from the robot, as explained by a participant:

Maybe if you are very lonely then you might want to talk about “I feel lonely, I do not know how to get in touch with someone, or I want someone to come and drink coffee with me”. But I think you want to talk about feelings. *(G2, P5, female)*

However, participants were mostly critical of the robot’s potential to reduce loneliness, because “it is a plastic thing” (*G3, P4, male*). They thought the robot could be beneficial to people who are completely alone, and may not have any other person to talk to, but not as something that other people would choose voluntarily:

I have a hard time seeing that, if the choice is to either talk to you or talk to the robot, that you would choose the robot. *(G3, P7, male)*.

Similar to the findings by Deutsch et al. (2019), the participants associated the robot as a social companion for people in their 90s, such as their parents, who spend a lot of time listening to audiobooks or watching television, as well as for people with dementia who may have limited possibilities for social interaction, similar to the findings by Khoo et al. (2023). For people at risk of disabilities, talking with a robot could help maintain cognitive skills similar to watching television. “Then this is also better than nothing, not hearing a voice perhaps for large parts of the day, you fill this in a sensible way and modulate the voice, so it sounds happier, friendlier, in such a situation” (*G3, P7, female*). In these situations, the robot is not expected to provide social companionship, but rather facilitate speaking or maintenance of cognitive skills:

I almost think it does not matter what (the robot) says, because if you’re alone, you eventually lose the ability to speak (−) And if you have not said anything, then the voice even starts to fade, and then when the children call once every six months, you are sitting all alone, then it is very important that there is someone who carries on a conversation, regardless of what it is says then. *(G4, P7, male)*

The robot facilitating voice usage could be leveraged as a recreational practice, such as singing:

In terms of voice, I would like to say that the most important thing you can do if you are alone is to walk around and hum and sing, so that your voice does not dry up again. And you might not think it’s so fun to do it yourself, but if the robot wanted to say, for example, “Which nursery rhymes do you remember?” or “Can you sing something?” or just activate both memory and voice. *(G4, P5, female)*

On the other hand, the fifth scenario (waking up to a new day) stimulated discussions around the robot being part of everyday activities and engaging in conversations similar to those they would have with any other person. These types of situations included, for instance, watching television together, discussing favorite programs, visiting museums together through virtual reality glasses, discussing the news or personal hobbies, such as stamp collection:

If you sit and watch a TV program, for example, and the robot sits along. Is it then able to hold a discussion about this program? (−) It can be something with more intellectual content. Is it then able to catch up and then be able to hold a discussion? *(G1, P8, female)*

### 4.7 Emotional expressiveness

In order to encourage older adults to share their feelings, the robot’s ability to convey emotions through empathetic responses and facial expressions was considered to be crucial:

It’s a lot about simply sharing feelings, being able to (show) happy and sad feelings and then it should be able to be responsive in some way and ask a lot of questions so you can talk out everything you feel. *(G1, P2, male)*

Is it possible (to share feelings with the robot)? Because I also thought about it if, for example, you come after a family visit and if someone has been ill, for example, and you are a little worried. Can the robot sort of show empathy and sort of give good advice like this, what to say if, well like this. something has happened. *(G1, P4, male)*

As such, the robot’s current voice and face were mostly considered to be ‘insensitive’ and ‘lacking emotions’, due to the lack of functionality to “laugh and smile” (*G3, P4, male*) and low variance in vocal intonation or facial expressions:

This voice that the robot has is very insensitive so it has no emotions in it, it just speaks very slowly. *(G2, P3, female)*

## 5 Design recommendations

Our prior work (Irfan et al., 2023) (among others described in Section 2.2) provides a starting point for using a foundation model (e.g., LLM) for a conversational companion robot for older adults. Deriving from the expectations of older adults outlined in the previous sections, and the challenges of LLMs encountered in our prior work based on the interactions with older adults, we offer actionable design recommendations for developing conversational companion robots that leverage foundation models, such as LLMs, vision-language models, and state-of-the-art architectures as their core, with potential relevance for other conversational robots and agents.

### 5.1 Passive and active listening

Passive listening, akin to a silent observer, would allow companion robots to discreetly gather invaluable insights from social events, creating a reservoir of knowledge to enhance future interactions of the user with their friends and family. On the other hand, active listening empowers the robot to actively engage in conversations with the user, asking relevant follow-up questions, and inspiring users to think critically about their perceptions and thoughts, in addition to improving trust in the robot (Anzabi and Umemuro, 2023). The key lies in the robot’s ability to discern the conversation’s context and choose the appropriate action–passive in social events and active when alone–ensuring a dynamic and meaningful dialogue that respects privacy and encourages thoughtful engagement.

For passive listening, relevant facts can be extracted from the dialogue during social events (e.g., friends gathering, family meetings) using LLMs through prompts, such as “summarize what we know about the user” (Irfan et al., 2023) and “How would you rephrase that in a few words?” (Scialom et al., 2022). In addition, retrieval-augmentation methods can be used for summarization (e.g., Xu et al., 2022). These facts can be stored in a knowledge base (e.g., user, friends, and family profiles) to use the learned information in conversation via paraphrasing, knowledge completion (Zhang et al., 2020), or construction (Kumar et al., 2020). Attention mechanisms can further improve the relevance of the extracted facts, especially when combined with multi-modal information, which is typically readily available in conversational robots (e.g., Janssens et al., 2022). These cues can help understand the situational context (social events or alone) to choose the appropriate listening strategy through activity, location, and event detection, which can be achieved through multi-modal foundation models (e.g., Afyouni et al., 2022; Fei et al., 2022), or more traditional methods, such as key-value, logic-based, or ontology-based approaches (Miranda et al., 2014).

Active listening can be achieved with LLMs through prompting, such as by describing the agent as an active listener that reflects on situations using shared history and follow-up questions (Irfan et al., 2023). In addition, LLMs can be combined with follow-up question generation mechanisms (S B et al., 2021; Ge et al., 2022). Fine-tuning on human-human interactions that contain follow-up questions, reflections, and inspirations to think positively can also increase the active listening capabilities of the agent (Khoo et al., 2023). These follow-up questions can be used to investigate the underlying aspects of the matters concerning the user’s loneliness, to increase their awareness of the root cause, and correspondingly address the problem. If, for instance, the cause is the lack of contact with family and friends, the user can be encouraged to reach out to them, similar to the ElliQ robot (Broadbent et al., 2024). While it is challenging to pinpoint the LLMs into certain directions, such use cases (e.g., loneliness, negative thoughts) for older adults can be pre-set in the system, in addition to topic detection via LLMs (Cahyawijaya et al., 2023) or other traditional methods (Ibrahim et al., 2018). Fine-tuning can also be used to trigger corresponding responses that could lead the dialogue model in the ‘right’ direction. In addition to follow-up questions, backchanneling can be used while the user is speaking, such as verbal acknowledgment (e.g., “uh huh”, “yeah”) and non-verbal gestures (e.g., head nods, smiles) to convey to the user that the robot is listening attentively (Johansson et al., 2016; Anzabi and Umemuro, 2023).

### 5.2 Lifelong learning and personalization

Unlike generic short-term interactions, forming companionship in everyday life requires learning knowledge about the user, which can encompass their family members, memories, preferences, or daily routines, as emphasized by older adults. Yet, merely acquiring this information is insufficient; it must also be effectively employed within context. This includes inquiring about the wellbeing or shared activities of specific family members, offering tailored recommendations aligned with the user’s preferences, referring to past conversations, and delivering timely reminders regarding the user’s schedule. This learning and adaptation cycle should be done continually over time, requiring long-term memory that scales gradually, without forgetting previously learned information, known as ‘catastrophic forgetting’ (Delange et al., 2021). Preservation of past knowledge and incremental learning of new information and adaptation is termed ‘lifelong (continual) learning’ (Thrun and Mitchell, 1995; Parisi et al., 2019). In comparison to ‘(reinforcement) learning from human feedback’ approaches, lifelong learning does not require explicit feedback in the dialogue and can be used to learn new facts from conversations, as well as update previously learned facts (Casper et al., 2023). While lifelong learning in foundation models showed benefits in various areas, such as question answering and empathetic dialogue generation (e.g., Scialom et al., 2022; Luo et al., 2023), open-domain dialogue is yet to be explored.

Learned facts in a conversation can be used to personalize the dialogue contextually, such as for providing reminders (similar to the ElliQ robot) and recommendations, adapting language style to be more personalized and suitable for older adults, and referring to a shared history. LLM prompts can be used to refer to these facts within the conversations (Irfan et al., 2023), in combination with retrieval augmentation and recommendation engines to provide personalized suggestions (see Chen J. et al. (2023) for a comprehensive survey on LLMs for personalization). Moreover, “in-context learning” and “chain of thought” (i.e., processing information step-by-step) reasoning (e.g., Wei et al., 2023) or planning can be used with conversation history for providing relevant recommendations (see Dong Q. et al. (2023) for a survey on in-context learning). LLMs can also be fine-tuned on a dataset of human-human interactions (e.g., older adults’ interactions in Khoo et al. (2023)) or based on human feedback (e.g., Ouyang et al., 2022) to improve the interaction style and personalize responses for long-term interactions.

Semantic understanding, i.e., the relations among entities within visual scenes through object, scene, or action recognition, can be achieved with foundation models to provide advice based on the situational context that extends beyond the capabilities of verbal context (Bommasani et al., 2022). For instance, the robot can suggest the user a recipe based on their preferences, and offer help with cooking verbally or potentially physically if integrated with manipulators, in which foundation models can be used for generating robot plans and actions, by referring to/using the learned locations of the equipment and ingredients (see Wang et al. (2024); Firoozi et al. (2023) for surveys of LLMs and foundation models in robotics for task planning and control).

In addition, the robot can be given a “persona” based on prompts that can evolve over time to maintain a believable and interesting character with its own preferences, opinions, and memories, which can help form the basis of a relationship with the user (e.g., Irfan et al., 2023; Landwehr et al., 2023).

### 5.3 Privacy preservation

Older adults often value their privacy and autonomy, and incorporating companion robots into their lives should be done with the utmost respect for these principles (Vandemeulebroucke et al., 2018). As these robots may gather and process personal information to enhance their interactions and functionality, ensuring robust privacy measures becomes imperative. Older adults may be more vulnerable to potential privacy breaches as they might not be aware of the span of information gathered in an interaction. Thus, companion robots should not compromise their sensitive data or personal preferences.

In order to prevent sharing personal information with others and to address the privacy concerns of older adults in a natural way (i.e., without requiring passwords or ID cards) in day-to-day interactions, a user recognition system can be employed on the companion robot. However, contrary to most approaches in face recognition, including foundation models, that require several images of users to be stored manually for pre-training (e.g., Yan et al., 2023), an architecture that can autonomously detect and gradually learn new users, known as ‘open world learning’, is necessary for real-world HRI (e.g., Irfan et al., 2021b; Belgiovine et al., 2022). Moreover, face recognition algorithms contain bias in identification (Irfan et al., 2021b; Buolamwini, 2023), and perform worse on older adults^9^. Thus, combining multi-modal information, such as age and gender, that decreases this bias is required to provide robust identification (Irfan et al., 2021b). Bias does not only affect user identification, but also appears in the form of misrepresentation (e.g., stereotypes), underrepresentation (e.g., lack of training data from a particular background), and overrepresentation (e.g., abundance of training data from a particular background that generates perspectives oriented towards them) in training data for foundation models, which can affect the performance between individuals from different backgrounds, lead to discrimination, and cause psychological harms (Bommasani et al., 2022; Weidinger et al., 2022).

Beyond the dangers of sharing information with other individuals, since foundation models require heavy computing, it is challenging to have embedded systems on robots, thus leading to cloud-based solutions, which carry the risk of sharing information with either the providers (e.g., OpenAI, Google) or cloud-services (e.g., Amazon Web Services) even when open-source models (e.g., LLaMA) are used. Thus, data should be anonymized when stored or passed to cloud services to prevent it from being used by third parties that train on user data or monitor it, by using privacy-preserving machine learning approaches (Xu et al. (2021) provide a review of such methods). In addition, cloud-based services open the floor for various types of hacker attacks that need to be addressed accordingly (Jia et al. (2023) review different types and suggestions to overcome them). Moreover, it should be made clear to older adults which data is stored and how, and who has access to it for transparency, which would improve trust in the robot (Berridge, 2015). Companion robots should not be used as surveillance systems by family members, care-takers, or governmental institutions, as that would break users’ trust in the robot, thus, decreasing shared social information and invalidating their purpose for social support.

Additionally, it is important to provide older adults with control of their own data by enabling the deletion of information verbally and easily, referred to as machine or knowledge “unlearning” (Bourtoule et al., 2020; Jang et al., 2022). Other ethical concerns for robots in elderly care are given by Vandemeulebroucke et al. (2018), and the risks posed by foundation models on privacy and corresponding solutions are discussed in further detail by Bommasani et al. (2022), Weidinger et al. (2022), and Zhang et al. (2023).

### 5.4 Information credibility and recency

Providing correct and factual answers is important for ensuring the robot’s credibility and dissipating concerns about deception (Berridge et al., 2023). A lack of correct information and awareness regarding news or political events renders the robot ineffective as a conversational partner. Moreover, users often combine their social and informational needs, making it convenient to engage the robot by posing practical yet informative questions about their daily schedule, weather updates, movie recommendations, fact-checking, or the latest news, similar to their use of spoken dialogue systems. More importantly, misinformation can be critical in health-related queries to the robot, especially for older adults who may be less inclined to independently fact-check such information, with medical foundation models yet to be sufficiently accurate (Yi et al., 2023). Providing explanations for contextual information inquired by the users require not only a correct understanding of the medical domain (Moor et al., 2023), but also explainable recommendations through prompting or fine-tuning (Zhao H. et al. (2023) provide a survey of explainability for LLMs).

The generation of text or responses that seem plausible but factually incorrect is referred to as “hallucination” in foundation models, which is a commonly recognized challenge (Weidinger et al., 2021; Irfan et al., 2023). Attention mechanisms, regularization techniques, retrieval-based methods, evaluating uncertainty in responses, memory augmentation, and rewards for increasing accuracy can help mitigate this challenge (see Ji et al. (2023) for detailed suggestions on these techniques). Additionally, anthropomorphism in foundation models can be deceptive for users (O’Neill and Connor, 2023), despite its benefits in likeability (Arora et al., 2021).

Furthermore, pre-trained models contain outdated information due to the limitations of their training data cut-off dates. This outdated information can be rectified by incorporating fact-checking mechanisms that use knowledge bases (e.g., Peng et al., 2023). Alongside lifelong learning for continuous fact updates, relevant facts can also be sourced from the internet, including real-time data like weather forecasts and news as requested by older adults, similar to spoken dialogue agents (e.g., Amazon Echo) and ElliQ robot, by utilizing LLMs with web browsing capabilities, such as Gemini^10^ and ChatGPT-4^11^.

It is also imperative to include strategies that mitigate adversarial behavior in users, in addition to the engraved toxic behavior in foundation models, such as the spread of misinformation and the generation of toxic, offensive, or undesirable responses. Such strategies include filtering (e.g., Dinan et al., 2019; Zellers et al., 2019; Schick et al., 2021), fine-tuning (e.g., Si et al., 2022), and user-based removal methods (Ju et al., 2022)). These measures are essential to safeguard the model’s factual accuracy and its adherence to the intended persona, thereby avoiding instances like Microsoft’s Tay when learning from users (Davis, 2016).

### 5.5 Social engagement

To alleviate loneliness among older adults, companion robots can provide users with the opportunity to reconnect with friends and family, thereby, mitigating the risks of over-reliance on interaction with technology. Foundation models capable of utilizing tools for social media, phones, and various devices (see Wang et al. (2024) for a survey) that leverage edge computing can enable this functionality (e.g., Dong L. et al., 2023; Shen et al., 2023). Additionally, robots can facilitate new online connections for users by harnessing their social media networks with the assistance of other deep learning architectures (e.g., Ding et al. (2017); Chen et al. (2020).

Conversational companion robots can enrich the communication and cognitive skills of older adults with dementia and at later stages of life (Cruz-Sandoval and Favela, 2019; Lima et al., 2022). LLMs can be prompted to encourage conversations in certain contexts and times of the day. In addition, cognitive games can be incorporated into the conversations or via tool use (e.g., phones). Fine-tuning on therapists’ interactions with such older adults can also shape the conversations toward incorporating these elements further into their daily lives. Furthermore, LLMs can be used to detect language impairment, which can help early diagnosis of dementia (Agbavor and Liang, 2022).

To maintain user engagement and interaction in daily life, conversations with companion robots should involve topics beyond the superficial small talk employed in current companion robots, such as ElliQ. The conversations should evolve around shared daily activities, hobbies, family, news, politics, and advice about situations. In contrast, the majority of conversations with LLMs tend to revolve around small talk, arising from a short number of turns and “let’s chat” approach used to obtain training data, which evidently results in small talk between humans (Doğruöz and Skantze, 2021; Irfan et al., 2023), which can be addressed through fine-tuning with real-world interactions. Moreover, they lack the ability to adapt to the dialogue context and maintain coherency with their limited memory, which can be overcome by memory augmentation. In addition, companion robots may be endowed with visual feedback in order to participate in the preferred leisure activities of the user that involve other media, such as watching television together and discussing programs or news. To enable such interactions, multi-modal foundation models (e.g., vision-language models) can be used to understand the content from images (e.g., Dai et al., 2023; Liu et al., 2023; Zhu et al., 2023), videos (e.g., Li K. et al., 2023; Maaz et al., 2023; Reid et al., 2024), and real-time interactions (e.g., Driess et al., 2023), which can be used in conversation.

### 5.6 Reflection of congruent emotions

Expressing empathetic responses in congruence with the emotional state of the users facilitates trust (Cramer et al., 2010), sustains long-term relationships with users (Bickmore and Picard, 2005), and improves likeability, especially for older adults (de Graaf et al., 2015), as supported by the perceptions of the older adults in our study. Empathy in dialogue can be conveyed through appreciation, agreement, and sharing of personal experiences (Lee et al., 2022), which can be achieved in LLMs that are shown to have high emotional awareness (Elyoseph et al., 2023). Prompting the model to be empathetic helps tailor its responses accordingly (e.g., Chen S. et al., 2023; Irfan et al., 2023). In addition, LLMs can be combined with supervised emotion recognition architectures (e.g. (Song et al., 2022)). Fine-tuning on empathetic dialogues between humans can guide the model toward providing appropriate responses (see Sorin et al. (2023) for a review of empathy in LLMs). Multi-modal affect recognition can also be used to dynamically adapt the emotion of the agent’s dialogue responses based on the emotions of users (e.g., Irfan et al., 2020; Hong et al., 2021).

In human-to-human communication, emotional prosody plays a more significant role than spoken words (Mehrabian and Wiener, 1967). In order for robots to sound emotionally expressive, as noted by the older adults in our study, “emotional voice conversion” (i.e., changing the emotion of the utterance) can be applied in text-to-speech (TTS) synthesis that allows variability in vocal intonation (see (Zhou et al., 2022) for a recent review). Recent methods have also incorporated LLMs into speech synthesis with emotional adaptation (Kang et al., 2023; Leng et al., 2023). Furthermore, Voicebox (Le et al., 2023) and ElevenLabs^12^ offer cross-lingual zero-shot TTS synthesis with emotionally appropriate vocal intonations.

Mimicking user expressions and behaviors, such as smiling and laughing with the user, can improve interpersonal coordination, boost interaction smoothness, and increase the likeability of the robot (Vicaria and Dickens, 2016). In addition, generating social signals that match the robot’s utterances can improve the believability, perceived friendliness, and politeness of the robot, and increase user interest in interacting with the robot (Sakai et al., 2012; Fischer et al., 2019). LLMs have also been incorporated into generating contextual facial expressions and gestures in virtual agents and robots via prompting (Alnuhait et al., 2023; Lee et al., 2023). Paiva et al. (2017) and Li and Deng (2022) give an overview of other methodologies for understanding, generating, and expressing emotions and empathy with robots and virtual agents.

### 5.7 Loneliness and social isolation

Design recommendations provided above were formulated by synthesizing older adults’ self-perceived expectations towards companion robots with the technical capabilities of foundation models. This study did not investigate whether or not these technical capabilities could fulfill older adults’ social needs or mitigate the experience of loneliness. Loneliness and social isolation are complex individual and societal phenomena, which are connected to other health-related issues and demographic changes in society. Loneliness is a subjective perception of a lack of social connectedness with social and personal relationships, communities and society (Newall and Menec, 2019), and it can be experienced regardless of the quality and quantity of social relationships (Kuoppamäki and Östlund, 2020). Therefore, not all older adults experiencing social isolation or lack of social contact necessarily consider themselves as lonely, and loneliness can be experienced regardless of the amount of social contact (Beneito-Montagut et al., 2018).

To investigate the association between open-domain dialogue with a conversational companion robot and experiences of loneliness in later life, future studies should explore older adults’ experiences with conversational companion robots outside the laboratory environment with a scale for measuring subjective perception of loneliness before and after interacting with the robot over time, such as UCLA Loneliness Scale (Russell, 1996) or Companion Robot Impact Scale (Broadbent et al., 2024). Loneliness and social isolation should be recognized as phenomena related to social and personal relationships and connectedness with the community, networks, and society. In this regard, the robot could function as a mediator between the user and their social networks to strengthen interpersonal ties between human-human relationships. By incorporating the technical capabilities presented above, conversational companion robots could be leveraged for social conversations that go beyond information retrieval towards more adaptive and personalized social conversations. These dialogues could proactively recognize the user’s perception of loneliness and guide the user with conversational exercises to increase user awareness of strategies to mitigate loneliness.

### 5.8 Study limitations

Our study has developed recommendations for designing conversational companion robots that leverage foundation models, focusing on LLMs for their dialogue capabilities, where we integrated older adults’ insights based on a co-design approach into tangible design recommendations. Rather than having the participants directly interact with the robot prior to discussions, we elicited participants’ expectations towards conversations based on visual design scenarios displaying the robot in diverse social contexts. Even though insights retrieved from this study represent participants’ shared expectations without having a direct interaction with the robot, this approach was a necessary step in order to learn about their needs and preferences, overcome current technological limitations, and be able to design more appropriate, inclusive, and accessible companion robots and dialogue models.

The participants’ cultural backgrounds may have influenced their views and expectations regarding the role and utility of robots in their daily routines, potentially differing from perspectives in other nations (Haring et al., 2014). Moreover, our thematic findings were based on the expectations of healthy older adults aged 66–86 years old, as such, these findings may not generalize to older adults beyond this age range or to individuals with cognitive impairments. While the chosen design scenarios captured common aspects of older adults’ daily lives, they did not encompass all possible scenarios, leaving room for diverse viewpoints that other situations may offer that can be discovered by deploying conversational companion robots at homes in long-term contexts. The focus group discussions elicited participants’ expectations of using the robot for social and emotional support, with a possibility to reduce loneliness among older adults. Therefore, the actual effects of whether or not the robot could mitigate the experience of loneliness remained unexplored in this study.

## 6 Conclusion

This study underscores the significance of aligning conversational companion robots with the distinct expectations and needs of older adults, aiming to provide social and emotional support in their daily lives. By involving older adults in the design process, we have gleaned invaluable insights into their desires for conversational companionship, ranging from active engagement during isolation to passive companionship in social settings, while prioritizing features like memory, personalization, privacy, information retrieval, social connectedness, empathy, and expressivity. Drawing from these findings, we provided recommendations on integrating foundation models, such as LLMs and vision-language models, and other state-of-the-art technology into conversational companion robots spanning key areas such as listening capabilities, lifelong learning, privacy safeguards, information credibility, social engagement, and congruent emotional expression generation through voice and facial cues. These insights offer a pivotal foundation not only for conversational companion robots, but also for the broader landscape of conversational agents that build upon foundation models.

## Data Availability

The original contributions presented in the study are included in the article/supplementary material, further inquiries can be directed to the corresponding author.

## Footnotes

1
https://www.statista.com/statistics/1133833/speech-to-text-transcript-accuracy-rate-among-leading-companies

2
http://www.parorobots.com

3
https://joyforall.com/

4
https://elliq.com/

5
https://www.acapela-group.com/

6 Videos of the design scenarios: https://youtu.be/jAk92vm3oEk

7
https://www.pexels.com/ and https://www.videvo.net/

8 Based on Furhat Robotics footage: https://youtu.be/jJ2N4PvAMos

9
https://nvlpubs.nist.gov/nistpubs/ir/2019/NIST.IR.8280.pdf

10
https://gemini.google.com/

11
https://chat.openai.com/

12
https://elevenlabs.io/text-to-speech
